# A Novel Screen-Printed Textile Interface for High-Density Electromyography Recording

**DOI:** 10.3390/s23031113

**Published:** 2023-01-18

**Authors:** Luis Pelaez Murciego, Abiodun Komolafe, Nikola Peřinka, Helga Nunes-Matos, Katja Junker, Ander García Díez, Senentxu Lanceros-Méndez, Russel Torah, Erika G. Spaich, Strahinja Dosen

**Affiliations:** 1 Neurorehabilitation Systems, Department of Health Science and Technology, Faculty of Medicine, Aalborg University, 9260 Aalborg, Denmark; 2 School of Electronics and Computer Science, University of Southampton, Southampton SO17 1BJ, UK; 3 BCMaterials, Basque Centre for Materials, Applications and Nanostructures, UPV/EHU Science Park, 48940 Leioa, Spain; 4 IDUN Technologies AG, 8152 Opfikon, Switzerland; 5 Ikerbasque, Basque Foundation for Science, 48009 Bilbao, Spain

**Keywords:** textile electrodes, high-density EMG, screen printing, myocontrol, amputation

## Abstract

Recording electrical muscle activity using a dense matrix of detection points (high-density electromyography, EMG) is of interest in a range of different applications, from human-machine interfacing to rehabilitation and clinical assessment. The wider application of high-density EMG is, however, limited as the clinical interfaces are not convenient for practical use (e.g., require conductive gel/cream). In the present study, we describe a novel dry electrode (TEX) in which the matrix of sensing pads is screen printed on textile and then coated with a soft polymer to ensure good skin-electrode contact. To benchmark the novel solution, an identical electrode was produced using state-of-the-art technology (polyethylene terephthalate with hydrogel, PET) and a process that ensured a high-quality sample. The two electrodes were then compared in terms of signal quality as well as functional application. The tests showed that the signals collected using PET and TEX were characterised by similar spectra, magnitude, spatial distribution and signal-to-noise ratio. The electrodes were used by seven healthy subjects and an amputee participant to recognise seven hand gestures, leading to similar performance during offline analysis and online control. The comprehensive assessment, therefore, demonstrated that the proposed textile interface is an attractive solution for practical applications.

## 1. Introduction

The use of electromyographic (EMG) signals for human-machine interfacing is rapidly increasing in popularity in both clinical and consumer applications [1,2]. Wet electrodes are still the golden standard for the recording of EMG from the surface of the skin [3]. In this approach, ionic gel or hydrogel is applied over the conductive pads to improve surface contact and reduce skin-electrode impedance [4]. Although “wet” electrodes can provide a signal of high quality, they face several challenges when considering their practical applications, especially during long-term monitoring: they need to be replaced after every use, the signal quality decreases over time and the cable connecting the pad to the amplifier is a recurrent source of movement artifacts. These issues become more pronounced as the number of recording pads increases, for instance, when using high-density EMG (HD-EMG). Commonly, an electrode for the recording of high-density EMG comprises a dense matrix of screen-printed conductive pads [5]. The electrode is attached to the skin by using a double-sided adhesive foam with holes arranged to match the design of the matrix [6]. The holes are then filled with conductive cream acting as an interface between the skin and the exposed pads. Only after such a preparation, the electrode can be applied to the skin. To facilitate the application of HD-EMG electrodes and make them more attractive for daily use, the development of solutions for dry recording has been in the focus of recent research efforts [7].

Dry electrodes are applied directly on the skin without conductive gel or cream, and this simplifies the setup by eliminating the need for electrode preparation and the cleaning of the skin after the recording [8,9]. The absence of cream or gel also avoids the creation of unwanted connections between the neighbouring pads due to spillover of the gel, which is a known issue, especially with multi-pad electrodes. Different technologies to produce dry surface electrodes have been proposed in recent years [7], with a special interest in textile solutions [10]. Textile electrodes are particularly suitable for practical application as they are reusable and can be integrated into breathable materials, thereby improving comfort and enabling long-term monitoring. Such electrodes are commonly produced using conductive yarn knitted [11,12,13] or embroidered [14,15] into the fabrics. However, conductive pads can be also created using standard screen-printing technology [16]. The advantage of screen printing is that the cables are substituted for tracks printed with conductive inks, thereby reducing motion artifacts and leading to a compact design. 

The use of screen printing to produce textile electrodes has been demonstrated in recent works [17]. In [18], the authors presented a single screen-printed textile electrode pad, which showed similar electrical properties compared to a hydrogel electrode, despite presenting an overall higher baseline noise. Recently, an electrode grid with multiple pads printed on textile was demonstrated in [19] and used to record EMG during physical activities, such as cycling, with promising results in terms of signal quality. Regarding the quality of the recording, the critical factor is the good contact between the electrode and the skin, which can be particularly challenging to achieve when using dry solutions [20]. For example, to overcome this issue, in [19], the authors had to apply individual 3D-printed plastic discs on the back of each conductive pad to improve contact between the electrode and the skin. Although functional, this solution complicates electrode production and is, therefore, not optimal for large-scale manufacturing.

In this study, we present a novel fully integrated solution for dry recording of HD-EMG based on a screen-printed textile electrode coated with a novel soft, polymer-based skin-electrode interface. We electrically characterised the novel solution and then demonstrated the feasibility of a functional application, namely, the use of the novel electrode for online gesture recognition. To benchmark the performance, we printed an identical electrode (i.e., the same pad size, shape and arrangement) using conventional technology and compared both electrical and functional results to that obtained using the novel prototype. 

## 2. Materials and Methods

Two identically configured matrix electrodes were produced using screen-printing technology: a polyethylene terephthalate substrate electrode with hydrogel (PET) and a textile-based dry electrode (TEX). The production process for both samples is explained in the next sections. 

### 2.1. Electrode Fabrication

#### 2.1.1. Textile Electrode

**Figure 1:**
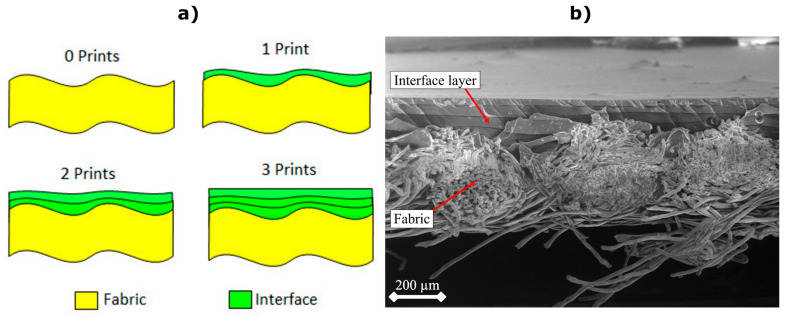
(**a**) The application of the primer layer to reduce the surface roughness of the fabric and improve homogeneity. (**b**) A cross-sectional micrograph obtained using scanning electron microscopy showing 4 primer layers printed on polyester cotton. The image shows the cross-section of 3 yarns, with other fibres running perpendicular. The boundaries between the 4 individually printed and cured primer layers can be seen filling the gaps in the fabric between the yarns and subsequently presenting a smoother top layer.

A woven textile provides greater comfort to the wearer compared to a non-woven due to improved drape and breathability of the fabric. However, the weave structure presents a series of voids and an uneven surface due to the typical gaps between the woven yarns that form the textile. With a simple passive print, such as a colour pattern, any voids may present aesthetic defects, but the overall pattern remains presentable. However, for electronic inks, due to the need for continuous electrical pathways, any printing defects can produce consequential short or open-circuit electrical failures. These defects lead to significantly reduced performance or complete failure of the printed electronic device. To achieve consistent prints on textile, a primer layer is first printed onto the textile to smoothen the surface [21], and the concept is shown in Figure 1. The primer layer is printed only where required for the subsequent electronic layers, therefore maintaining the maximum original properties of the fabric and minimising the physical impact of additional electronic layers [22]. 

**Figure 2:**
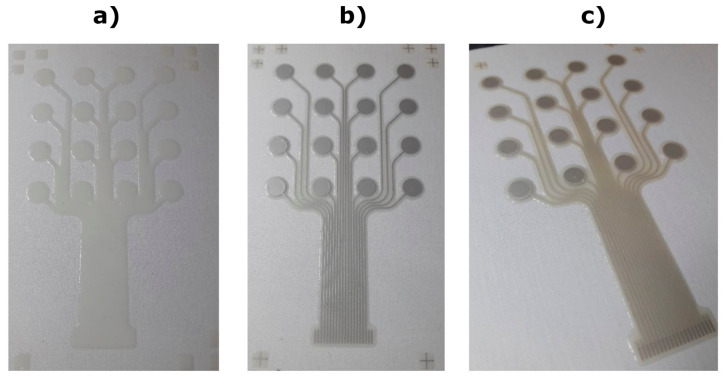
The printed electrode stages when printing on a polyester cotton woven textile. (**a**) Primer layer. (**b**) Conductive layer. (**c**) Encapsulation layer. The electrode pads remain exposed so that the subsequent skin-electrode interface material can be applied.

The printed electrode structure consists of a primer layer (Smart Fabric Inks Ltd.—Fabinks UV-IF-1004—polyurethane primer), conductive tracks (Fabinks TC-C4007—silver conductor) and encapsulation (Fabinks UV-IF-1004). The fabric base was a 65% polyester, 35% cotton blend (OpticWhite A1656—Whaley’s Bradford, UK), a thin standard fabric (twill 2 × 1 weave) that was previously identified by the authors as suitable for screen printing [23]. The electrode design comprises a 4 × 4 layout of 16 pads of 6 mm diameter and 12 mm distance between the pads. The leads extend for 50 mm from the bottom row to the end terminals and were grouped together with 1 mm pitch to provide compatibility with standard connectors. The primer and encapsulation layer closely follow the electrode pattern to maximise the breathability of the fabric. The encapsulation layer provides electrical insulation for the conductive tracks to prevent any short circuits between the pads, and the encapsulation pattern leaves the silver circular pads and the connector area at the bottom of the pattern exposed to allow the electrical connection. Figure 2 shows the three printing stages and patterns. 

All the layers were printed using a DEK248 semi-automatic screen-printer. The primer layer was printed directly onto the fabric with 4 layers, where each layer was individually printed and then cured via a 400 W Fe UV bulb (UV-Light Technology Ltd., Oldbury, UK) for 30 s (~1500 mJ/cm^2^). The silver conductor layer was printed with 1 layer and cured at 120 °C for 15 min. Finally, the encapsulation layer was printed with 1 layer and cured via the same UV process as the primer layer. Once the screen printing was complete, the skin-electrode interface was applied to the electrode pad areas as described later.

- Surface roughness measurements

**Figure 3:**
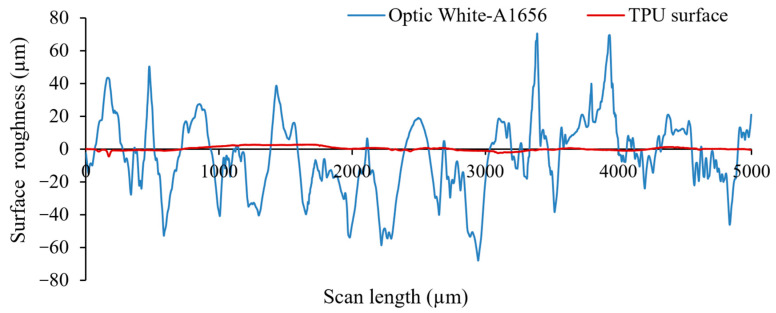
A comparison of the surface profiler measurements for the bare fabric substrate (blue line) and the screen-printed thermoplastic polyurethane (TPU) primer layer on top of the fabric (red line).

To ensure the surface roughness of the fabric was sufficiently reduced, a 2D Tencor P-11 surface profiler was used to measure the profile of the primer layer after printing on the fabric before the subsequent silver conductor layer was printed. A profile was measured with 10,000 profile points over a scan length of 5 mm. Figure 3 shows the comparison of the roughness between the surface profile for bare fabric and the top surface of the 4 printed layers of the primer. 

The fabric has an average roughness of 17.4 ± 23.1 µm, while with the primer layer, this is reduced to just 0.8 ± 1.22 µm, presenting a smoother and more homogenous surface to print the conductive layer, as well as providing all-around encapsulation protection from sweat or water ingression during washing.

- Electrode-skin interface

The dry skin-electrode interface, IDUN DRYODE™ Ink, used in this study is based on a soft, flexible, medical-grade polymer. The material is highly conductive, while the flexibility provided by the polymeric matrix is retained. To fabricate flat pads, the IDUN DRYODE™ Ink was deposited onto the conductive pad by doctor blading. The viscosity of the IDUN DRYODE™ ink can be modified within a range of 60 mPa∙s and 200 mPa∙s to adapt to other industry methods, including screen printing or dip coating. 

To coat the textile-based electrodes with the dry interface, an exact stencil of the electrode layout was designed in Inkscape and cut out from a 0.2 mm thick, self-adhesive stencil foil with a Curio cutting plotter. The stencil was then aligned with the printed textile electrode and pressed onto the surface firmly. The textile electrode with the stencil was fixated on an even surface with masking tape. Afterward, a suitable quantity of the IDUN DRYODE™ Ink was applied to one side of the stencil foil and then distributed evenly across with a steel doctor blade. The stencil was then removed, and the textile electrode was fully dried in an oven at 60 °C. This process can be repeated if multiple layers of the skin-electrode interface are required. For this study, we applied two layers of the dry interface to the textile electrode, which provided a small protuberance to improve the contact surface with the skin.

#### 2.1.2. PET Electrode

**Figure 4:**
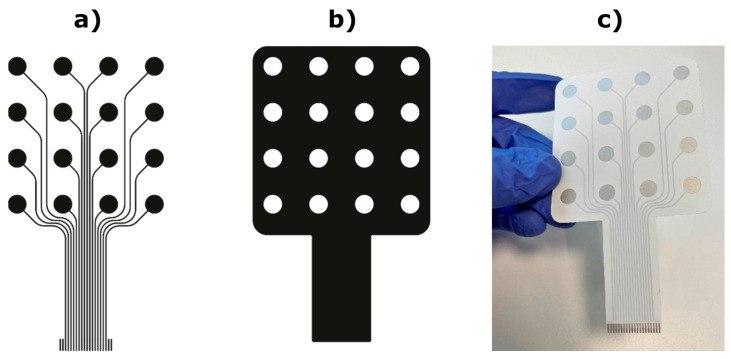
Design of the printed flexible PET electrode. (**a**) Design of the silver electrode pads with leads. (**b**) Design of the dielectric protective layer. (**c**) Printed flexible electrode.

The PET recording electrodes were printed using the semiautomatic screen-printing machine DX3050D (Shenzhen Dstar Machine, Hong Kong) on polyethylene terephthalate (PET) flexible transparent foil (Melinex^®^ ST 506, DuPont Teijin Films, Dumfries, UK, thickness 125 µm). The design used the same electrode layout as the TEX substrate but without the additional primer layer. First, three passes of silver ink (DuPont 5065 silver conductor) were printed directly on the PET substrate, followed by the printing of two passes of the dielectric ink (DuPont ME779), which was used for the encapsulation and protection of the printed leads (Figure 4). After each printing pass, the printed layers were first dried at 90 °C in a hot air oven (J.P. Selecta Digitronic-TFT) for 5 min. Finally, the printed devices were cured at 120 °C for 20 min to complete the sintering of the printed silver electrodes. The thickness of the dielectric and the silver printed layer was determined using a digital micrometer Mutitoyo to be 12 ± 1 µm and 18 ± 1 µm, respectively. For the used conductive silver ink DuPont 5065, the electrical conductivity of 5.6 × 10^4^ S cm^−1^ was measured using a two-point probe and a digital multimeter Amprobe AMP-510-EUR. Before using the electrode, the exposed pads were covered with identically shaped discs of conductive hydrogel (AG702, Axelgaard, Lystrup, Denmark).

- Scanning electron microscopy characterization

**Figure 5:**
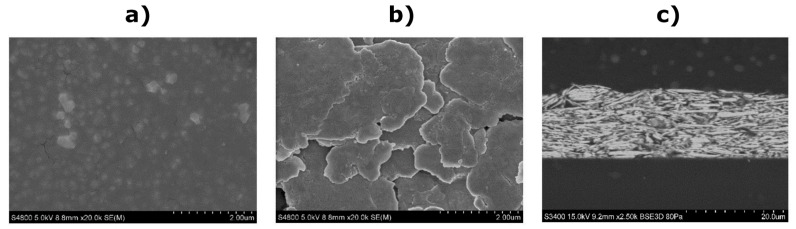
SEM images of the printed EMG electrode surface. (**a**) White dielectric layer (scale of 2 µm). (**b**) Silver electrode pad (scale of 2 µm). (**c**) Cross-sectional image of the printed silver electrode pad (scale of 20 µm).

The scanning electron microscopy (SEM) images were acquired with a Hitachi S-4800 and Hitachi S-3400 set-up at 5 kV and 15 kV, respectively. The samples were metalized with a thin sputtered layer of gold. As can be observed in the representative SEM images shown in Figure 5, the surface of the printed dielectric layer is formed by well-distributed sub-micrometre dielectric particles embedded in a polymer with a relatively smooth surface. On the other hand, the silver conductive layer is formed by silver flake-type particles with a size exceeding 1 µm in most cases, which are densely stacked to a compact layer (as shown in the cross-sectional image in Figure 5c).

- Atomic force microscopy measurements

**Figure 6:**
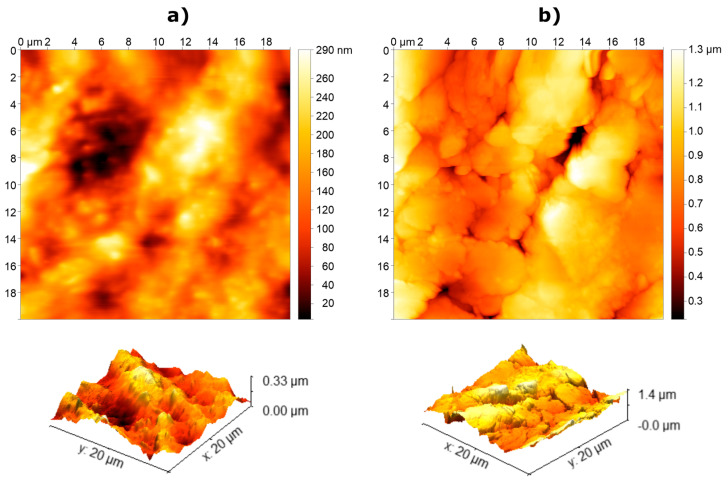
AFM surface measurements (2D and 3D topography) of the printed electrode surface. (**a**) White dielectric layer. (**b**) Silver electrode pad.

The surface roughness of each printed material was further analysed by means of atomic force microscopy (AFM). The surface topography roughness of the printed layers was assessed by means of AFM in the tapping mode with a Nano-Observer AFM (CS Instruments, Paris, France), and the measurements were later evaluated using Gwyddion software. The measured AFM data were treated using the line-by-line second order polynomial levelling. The measurements were carried out over an area of 20 × 20 µm and afterward used to calculate the root-mean-square (RMS) roughness (σ) as $\sigma =\sqrt{\frac{1}{N}\;\overset{N}{\underset{i=1}{\sum }}{\left(z_{i}-\;\bar{z}\right)}^{2}}$, where z_i_ is the height measured at each point and $\;\bar{z}$ is the mean value of the area. The estimated σ was 0.06 µm for the printed dielectric layer and 0.20 µm for the printed silver electrodes, respectively. Figure 6 shows the 2D and 3D topography images of the dielectric and silver layers. The scale in the 2D images was shifted using Gwyddion software so that the minimum value was near zero. Additionally, the scale of the 3D images was adjusted to each sample for better visualization and, therefore, cannot be directly compared. The resulting roughness values of the printed layers are significantly below the values typical for the human skin (~10 µm), and, therefore, good contact between the electrode and the skin should be ensured [24].

### 2.2. Electrode Assessment and Comparison

**Figure 7:**
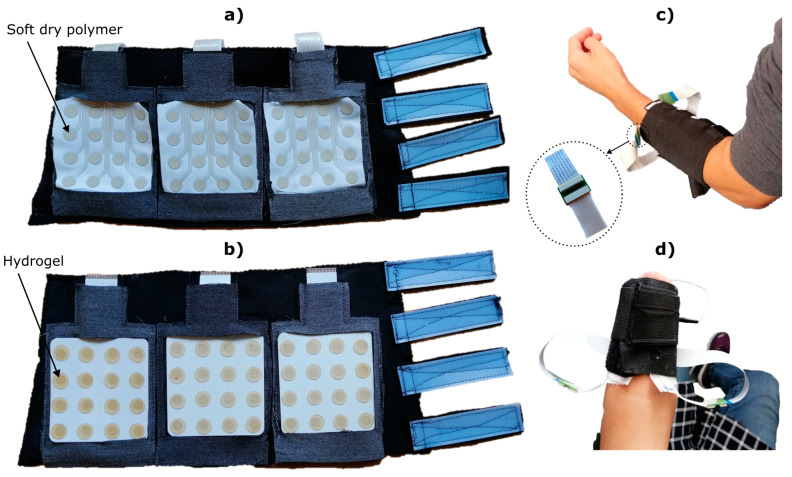
Sample armbands used to record EMG signals for offline analysis and online control. (**a**) Three TEX matrices placed in the garment. The exposed pads of the matrix were coated with a highly conductive soft polymer, as described in Section 2.1.1. (**b**) Three PET matrices placed in the garment. Round hydrogel discs were applied to each recording pad as electrode-skin interface. (**c**) An able-bodied participant wearing the armband. Encircled is a detail view of the connection between the textile and the flexible cables using a double-sided ZIF adaptor. (**d**) Transradial amputee wearing a custom-made elastic armband with the TEX electrodes adapted to the size of the residual limb.

A custom-made garment was designed to facilitate the application of the electrodes (Figure 7). The garment integrated three pockets to introduce and secure the electrode matrices. The pockets allowed the electrodes to be easily exchanged, and they ensured an even spacing between the matrices during the recordings. The garment was made using elastic fabric to ensure a tight fit and, hence, good contact between the pads and the skin, and it was equipped with hook and loop fasteners to adapt to different arm sizes. However, if the arm size of the participant was below the minimum length of the garment, the electrodes were attached using a standard elastic band (Figure 7d).

The EMG signals were recorded using a multichannel biomedical amplifier (Quattrocento, OTBioelettronica, Turin, Italy). The electrode grids were connected to the amplifier using a custom-made adaptor placed in a dedicated pocket on the backside of the garment. To facilitate the setup, the printed tracks of the fabric matrices were extended using commercial flat flexible cable (WR-FFC 1 mm). The screen-printed tracks were then attached to the flat cables through a double-sided adaptor using ZIF connectors (Amphenol TE 2-84981-0, 20 pins, 1mm pitch). The signals were band-pass filtered using built-in filters (2nd order Butterworth, bandwidth 10–500 Hz) and sampled at 2048 Hz.

Seven able-bodied subjects aged between 25–35 years and one subject with transradial amputation (49 years) participated in the experiment. The amputee lost her left, non-dominant hand in a traumatic amputation, at approximately two-thirds of the forearm below the elbow joint, 10 years ago. The functionality of the elbow joint was intact. The amputee has been using a single degree-of-freedom prosthesis with 2 EMG electrodes and, hence, did not have previous experience with pattern classification.

The study was conducted following the Declaration of Helsinki and was approved by the North Denmark Region Committee on Health Research Ethics (20190036). The participants received oral and written information before the beginning of the experiment and signed the informed consent form. 

During the recording session, the participants sat comfortably in a chair in front of a computer screen. The skin was carefully shaved and cleaned using abrasive paste (everi, Spes Medica, Genoa, Italy) before placing the electrodes. Three electrode grids were inserted in the garment pockets, as shown in Figure 7, and the garment was strapped around the forearm using the hook and loop straps, at approximately 20% of the forearm length distally from the elbow crease. The participants performed the full experimental protocol using both electrode types (TEX and PET), and the order of the electrodes was pseudorandomized across subjects. After the protocol was completed with the first electrode type, the garment was removed and replaced with another garment containing the other electrode type. The electrode location was carefully marked on the skin with a marker to ensure the same positioning. The experimental protocol included two phases, namely, (1) the recording of EMG data for offline analysis and classifier training and (2) online control task, as explained below. The online task consisted of recognizing seven hand gestures, namely, “power grasp”, “one-finger pinch”, “three-finger pinch”, “lateral grasp”, “index pointing”, “hand open” and “rest”. For the amputee subject, the classification task was reduced to six classes (all classes except “three-finger pinch”), as a quick pilot test demonstrated that it was challenging for her to produce “three-finger pinch”.

#### 2.2.1. EMG Recording

A graphical user interface guided the participants during data collection. Firstly, the maximum voluntary contraction (MVC) was measured for each gesture. The participants were asked to maintain an isometric contraction at the maximum, but still comfortable intensity, for three seconds. The MVC values were calculated for each gesture as the root mean square (RMS) of the EMG signal computed in 250-ms windows over the contraction period and averaged across all the channels. 

To collect the EMG data for classifier training and offline analysis, the participants performed two consecutive isometric contractions for each gesture at two intensity levels (30% and 60% MVC). The momentary contraction level was provided as online visual feedback to the participants, and they were asked to match the target activation profile. The target profile comprised two trapezoidal shapes, separated with a 5-s rest period. Each trapezoid included a 1-s ascending/descending slope and a 4-s plateau placed at 30% and 60% MVC for the first and the second trapezoid, respectively. Different gestures were performed sequentially in a pseudorandom order with fifteen seconds pause between each gesture. The data was segmented to include only the EMG generated during the contractions (RMS EMG > 10% MVC). Five time-domain features that are commonly used for gesture recognition were extracted from the segmented data using 250-ms sliding windows with 50% overlap [25], namely, mean absolute value (MAV), zero crossing (ZC), slope sign change (SSC), wavelength (WL) and the logarithm of variance (LogVar). The resulting dataset was randomly divided into training (70%) and testing (30%) using a stratified hold-out method and used to train a classifier based on linear discriminant analysis, which is a commonly used method for myoelectric control [26]. 

#### 2.2.2. Online Control

The online evaluation was implemented using a gesture-matching task. The participants were asked to perform a target gesture shown on the computer screen, while they simultaneously received visual feedback about the gesture predicted by the classifier. The task was considered successful if the participant maintained the target gesture continuously for 2 s (16 decisions) with an accepted error of two misclassified decisions to minimise the flickering impact on the control stability. Similarly, the amputee participant was asked to generate the target gesture for 1 s (10 decisions) with an accepted error of two misclassified decisions. The maximum time to complete the task was 10 s, and, after this time, the task was considered as failed. The target gestures were presented in a pseudorandom sequence with 15-s rest between successive presentations.

### 2.3. Data Analysis

#### 2.3.1. EMG Signal Quality

The MVC values were used as the standardised benchmark to compare the magnitude of the signals recorded by the TEX and PET electrodes. A Bland–Altman plot was used to evaluate the agreement (95% limits of agreement) between the PET-TEX MVC measurements. The average signal-to-noise ratio (SNR) was calculated as $\mathrm{SNR}=20\text{ × }log_{10}\left(\frac{{\mathrm{EMG}}_{\mathrm{MVC}\;}}{{\mathrm{EMG}}_{\mathrm{REST}\;}}\right)$ across gestures, where ${\mathrm{EMG}}_{\mathrm{MVC}}$ was the mean MVC calculated for each subject and movement class and ${\mathrm{EMG}}_{\mathrm{REST}}$ was the mean RMS of EMG obtained during rest. The power spectral density (PSD) of the EMG signal was computed from one representative subject using the Welch method averaged across pads, using data from the entire session during contractions (EMG activity) and during rest (baseline noise). In addition, the quality of contact between the electrode and the skin was estimated using the lead-off detection system integrated into the amplifier device. The use of lead-off detention is not recommended to provide an accurate value of the skin-electrode impedance [27,28]. Hence, in the present study, we use it only to compare the quality of skin-electrode contact between the two interfaces in one representative subject after wearing each armband setup for 20 minutes to reduce the variability of the measurements [20]. The impedance was measured for each pad at a frequency of 250 Hz and averaged across all channels. Additionally, the correlation coefficient was used to measure the similarity between the spatial patterns of muscle activity for each subject recorded using both interfaces. The coefficient was calculated between the sequences of averaged RMS values (one value per pad) corresponding to the contraction period recorded using TEX and PET. The coefficient was determined for each gesture, and then the overall average was calculated for each subject.

#### 2.3.2. Offline and Online Classification Performance

The offline classification accuracy was computed by applying the trained classifier to the testing dataset. The accuracy was defined as the number of correctly classified test samples divided by the total number of test samples. Additionally, three outcome measures [29] were used to evaluate the online control: (1) completion rate, (2) completion time and (3) the number of oscillations. The completion rate was computed as the number of successfully completed trials over the total number of trials. The completion time was measured as the time interval from the moment the target gesture was shown on the screen to the moment the subject successfully accomplished the task. The number of oscillations was calculated as the number of times the classifier prediction changed during the successful trials. The normality of the data was assessed using the Shapiro-Wilk test. As the data were normally distributed, repeated measures analysis of variance (RM-ANOVA) was used. Paired *t*-tests were conducted for pairwise comparison and adjusted for multiple comparisons with Bonferroni correction. The significance threshold was set at *p* < 0.05. 

## 3. Results

### 3.1. EMG Signal Detection

**Figure 8:**
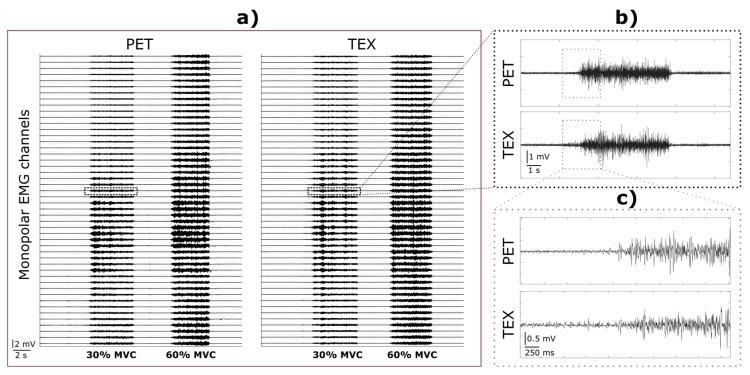
EMG signals from a representative participant recorded while performing a “power grasp” gesture. (**a**) Monopolar signals recorded from 48 channels using PET (left) and TEX (right). The dashed line marks two EMG bursts corresponding to the muscle contractions at 30% MVC from a selected representative channel. (**b**) The two selected contractions recorded by PET (top) and TEX (bottom). (**c**) The initial phases of the two selected contractions recorded using PET (top) and TEX (bottom).

Figure 8 illustrates the EMG signals recorded from a representative healthy participant during the execution of a power grasp using PET (Figure 8a, left) and TEX (Figure 8a, right) electrodes. Both recordings are characterised by a flat baseline (no motion artifacts), clear EMG bursts, and a similar signal magnitude and activity pattern across the channels and within single bursts (Figure 8b). Overall, the mean SNR across channels for PET and TEX was similar, i.e., 42.39 ± 8.41 dB vs. 44.42 ± 7.2 dB at 30% MVC and 51.9 ± 7.25 dB vs. 57.9 ± 5.71 dB at 60% MVC, respectively. 

**Figure 9:**
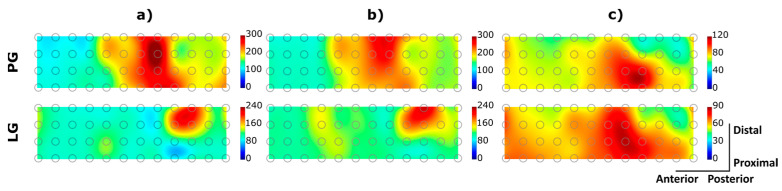
Spatial pattern of muscle activity recorded from a representative able-bodied participant wearing the (**a**) PET and (**b**) TEX interface and (**c**) the amputee subject wearing the TEX interface. The correlation coefficients calculated between PET and TEX for the PG maps and LG maps of the able-bodied participant were 0.88 and 0.92, respectively. The units for the colour scale are µV. (PG: power grasp, LG: lateral grasp).

**Figure 10:**
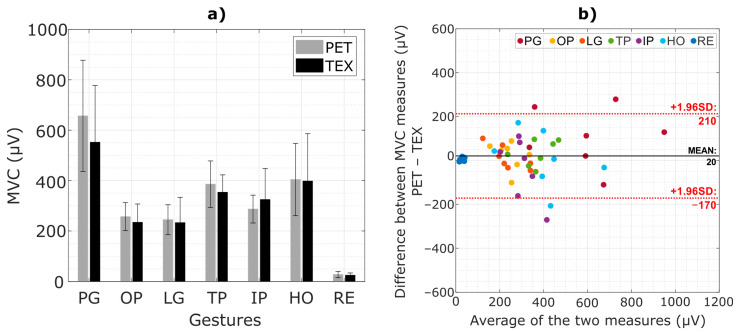
(**a**) Averaged MVC values (mean ± std) across participants grouped by gestures. (**b**) Bland-Altman plot of the MVC measures for all participants. Each colour represents a gesture. A solid line corresponds to the bias and red dashed lines indicate the limits of agreement. (PG: power grasp, OP: one finger pinch, LG: lateral grasp, TP: three fingers pinch, IP: index pointing, HO: hand open, RE: rest).

Figure 9 illustrates the interpolated maps of spatial muscle activation from a representative able-bodied participant and the amputee participant. Both participants performed two gestures (i.e., “power grasp” and “lateral grasp”) at 30% MVC while wearing PET (Figure 9a) and TEX electrodes (Figure 9b,c). In the able-bodied participant, the peak areas of muscle activity were concentrated in the volar matrices, which corresponds to the activity of the flexor muscles. In addition, the spatial patterns of muscle activity characterizing the two grasp types are clearly different. Some differences also exist in the amputee participant, but they are much less expressed, and the muscle activity elicited during lateral grasp was more diffuse. There are small variations in the maximum amplitudes recorded using the two electrodes, which reflect the differences in the MVC values shown in Figure 10. However, the shape and location of the EMG “hot spots” were rather similar between the electrodes. In the amputee participant, the main area of activation was located more medially when performing the power grasp. The overall correlation coefficient (mean ± std) calculated between muscle patterns obtained using PET and TEX across gestures and participants was 0.78 ± 0.19.

Figure 10a shows the average MVC values (mean ± std across all subjects) recorded using both electrode types. The obtained MVC values were similar for the two interfaces consistently across the gestures, with the largest difference for the power grasp. The baseline noise corresponding to the EMG signal during rest was similar between the two electrodes (27.8 ± 12.8 µV for TEX and 25.05 ± 8.9 µV for PET). The average SNR across participants was 53.2 ± 9.6 dB using PET and 52.7 ± 7.2 dB using TEX. There were no statistically significant differences between TEX and PET in any of the aforementioned measures.

Figure 10b shows the Bland-Altman plot of the MVC measures using PET and TEX. There are two outliers for the class “power grasp”, one for “hand open” and one for “index pointing”. Overall, most of the points are contained within the limits of agreement and are evenly distributed around the mean with a minor positive bias (20 µV) between PET and TEX measurements.

**Figure 11:**
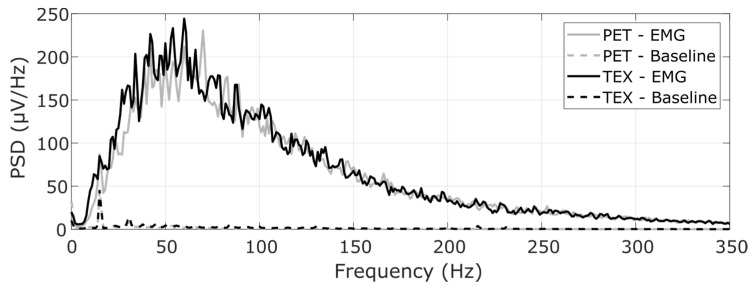
Mean power spectral density of the EMG recorded from a representative participant. Solid lines correspond to the mean PSD across channels calculated from the EMG signal recorded during muscle contractions, while the dashed lines represent the mean PSD during rest. The standard deviation was similar between interfaces and hence not shown to improve readability.

Figure 11 shows the mean PSD calculated across the full grid of channels from a representative subject. Both electrodes produced similar PSD profiles, with most of the power concentrated in the range of 10–250 Hz, which corresponds to the expected bandwidth of the surface EMG. The PSD of the baseline noise was at least two orders of magnitude lower compared to that of the EMG signal. Moreover, the impedance measured (mean ± std) across pads was similar between TEX (5.6 ± 3.3 kΩ) and PET (3.9 ± 4.8 kΩ).

### 3.2. Gesture Recognition

**Figure 12:**
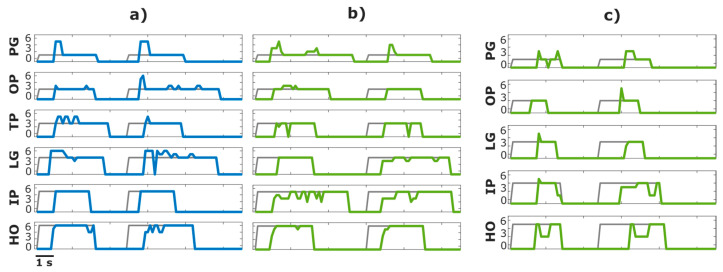
Illustration of the online classification task. The grey lines indicate the target gesture while the coloured lines denote the estimated class in an able-bodied participant using (**a**) PET and (**b**) TEX and (**c**) the amputee participant using TEX. Each row corresponds to a different gesture, and it includes two trials separated by a break. The movement classes are indicated by numbers: 1, 2, 3, 4, 5 and 6 correspond to the power grasp (PG), one finger pinch (OP), three finger pinch (TP), lateral grasp (LG), index pointing (IP) and hand open (HO), respectively.

The performance of offline classification was almost perfect for both PET (99.7%) and TEX (99.3%). The amputee participant achieved a similarly high offline performance (96.9%). Figure 12 illustrates the online control task. The grey line indicates the moment in time the target gesture was shown on the computer screen, while the coloured lines denote the classifier output (estimated gesture). The able-bodied participant completed most of the trials (11 out of 12) using both electrode types. A delay between the prediction and target profile corresponds to the reaction time of the participant. Once the participant started contracting, he produced and maintained the target gesture with few oscillations (i.e., wrong estimations). Similarly, the amputee participant was able to complete most of the trials with few oscillations (Figure 12c).

**Figure 13:**
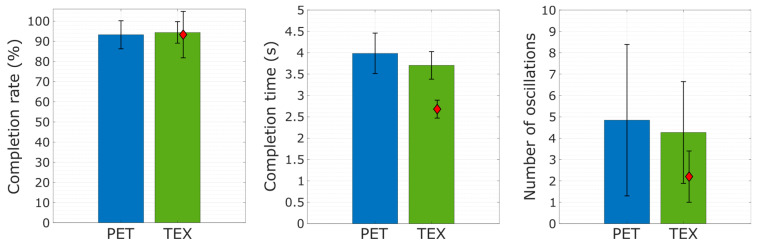
The outcome measures for the online gesture-matching task. The bars represent the mean and standard deviation for the completion rate, completion time and the number of oscillations obtained in able-bodied participants. The results for the amputee subject are denoted with a diamond.

The summary results (mean ± std across subjects) for the online task are shown in Figure 13. The participants achieved a similarly high completion rate with both electrode types (94.4 ± 5.33% using TEX and 93.25 ± 6.95% with PET). Moreover, there was no statistically significant difference between TEX and PET in completion time (3.7 ± 0.32 s vs 3.98 ± 0.97 s) or the number of oscillations (4.2 ± 2.3 vs 4.8 ± 3.5). The amputee participant achieved a completion rate (mean ± std across trials) of 93.3% ± 11% and a completion time of 2.68 ± 0.2 s.

## 4. Discussion

We have presented a novel textile interface for the recording of a high-density EMG from the forearm muscles. The interface comprises three screen-printed electrodes with 48 pads integrated into a flexible garment and coated with a dry formula ensuring good skin-electrode contact. The presented solution is, therefore, convenient for practical applications due to simple setup as well as mass production thanks to screen printing. We have conducted a set of tests to comprehensively assess the novel solution by comparing it to the benchmark. Importantly, the benchmark was a conventional electrode printed on PET film, designed from scratch (Figure 4) to have an identical configuration as the TEX and created using a carefully developed procedure that ensured a high quality and rigorous assessment of the produced sample (Figure 5 and Figure 6). Such an approach guaranteed a sound benchmarking of the novel technology. Typically, the textile designs presented in the literature are described and demonstrated, mostly using offline analysis [11,15,19,30], and they are rarely compared to the equivalent conventional solutions [15,18], as in the present study. 

The results demonstrated that the dry fabric matrices provided signals of similar quality as those acquired using the conventional state-of-the-art electrode (PET electrodes covered with hydrogel). The two solutions were characterised by similar signal-to-noise ratio, frequency spectra and spatial distribution of the recorded EMG signals. They also enabled similar performance in offline gesture classification as well as during online gesture-matching tasks. The comparison of power spectrum densities of EMG signals recorded using both electrodes revealed almost identical characteristics, with most of the frequency components contained within the 10–250 Hz band. The estimated SNR and frequency spectra demonstrated that the fundamental electrical properties of the two interfaces were indeed similar when analysed as an overall average (across channels). The Bland-Altman plot analysis revealed a minimum bias between PET-TEX MVC measures. The good correlation across pads additionally showed that the novel solution can correctly capture the spatial distribution of the muscle activity around the forearm, which is of interest in the biomechanical analysis [31] as well as in myoelectric control [32,33]. The obtained correlation of ~0.8 should be considered in light of the natural variability that exists in EMG patterns when performing different repetitions of the same gesture. Finally, the classification results confirm that the textile matrix can be used for human-machine interfacing, where users’ gestures are detected and translated into commands for an external device (myoelectric control). Importantly, the myoelectric control has been evaluated also during the online control task, which represents a more relevant assessment compared to offline classification [34]. This evaluation has been performed on a person with an amputation, who is a potential user of the novel technology in a clinical context (i.e., textile interface embedded into a socket for prosthesis control). 

Only a few studies in the literature describing textile solutions included online assessment, and none of them tested an amputee participant. The online control results showed that the amputee completed the tasks with high accuracy (93.3% ± 11%) when wearing the TEX electrodes garment, and this accuracy was similar to that of able-bodied participants using TEX (94.4 ± 5.33%) and PET (93.25 ± 6.95%). The amputee was naïve regarding pattern classification, and the training session was relatively short (i.e., below 15 min). However, the number of classes was reduced by one for the amputee (six classes), as an initial test demonstrated that it was difficult to control seven classes. In line with this, the activity map (Figure 9c) showed similar muscle activation patterns for two different gestures. This probably reflects the reduced dexterity of the amputee due to fewer and/or less active muscles in the forearm as a result of the surgery.

These preliminary results are therefore encouraging for future clinical use of textile garment electrodes, especially when high performance is combined with ease of application. After wrapping the garment around the forearm and attaching the straps, the electrode is ready for recording. Although such a scenario was not investigated in the present study, these characteristics make the proposed solution very attractive for future prospective use in rehabilitation, where clinical personnel work under strict time constraints (e.g., 30 min rehab sessions), and the time to set up, therefore, needs to be minimised. 

The TEX electrode maintained good contact with the skin while the subjects performed hand gestures, as indicated by the absence of artifacts in the raw EMG recordings during the onset and offset of the movements (Figure 8). The good signal stability during the execution of the gestures can be explained by the mechanical buffer provided by the protuberant soft polymer that can absorb small skin deformations produced by the volume changes in the undelaying muscles during contractions. The presence of hydrogel in the PET electrode produced a similar cushioning effect, avoiding breaks in contact between the electrode and the skin during hand movements. However, hydrogel must be moisturized regularly to maintain stickiness and ensure adherence as well as electrical conductivity. Furthermore, hydrogel peels off easily, it wears out over time and can dry out permanently if is not carefully maintained. For this reason, and despite its good electrical properties, the hydrogel is not a suitable option for wearable solutions or long-term monitoring. On the other hand, the soft polymer used in our TEX electrode is enduringly adhered to the conductive pads and does not require further maintenance or preparation. 

Gesture recognition results were on par with the results presented in the literature using wet [35,36] and dry electrodes [11,13,15,30,37,38]. Our electrode integrated substantially more pads compared to most textile solutions presented in the literature. A dry matrix with similar pad density was described in [37], but this was a semi-rigid solution design based on printed circuit boards. The 32 conductive pads were made of gold dipped discs, and each pair of pads was encapsulated into small rigid cases that were held together using a flexible substrate. Low-cost embroidered electrodes were described in [15] using four pairs of electrodes that were evaluated offline. In [30], the authors presented a high-density sleeve with 100 knitted electrodes using steel yarns to measure signals from the upper arm and forearm muscles. The same approach was used in [38], where the authors presented a wireless textile armband for real-time myoelectric control with eight bipolar knitted electrodes. Similarly, in [11], the authors produced a sensor band placed around the forearm with eight knitted electrodes using intarsia pattern knitting, with a combination of silver-plated and nonconductive polyester yarn. In [13], the authors produced a textile sleeve with seven bipolar electrodes knitted with silicon-based conductive yarn. Importantly, most of the embroidered and knitted solutions mentioned above were connected to the amplifier through snap connectors mounted on the back of each recording pad. This approach requires the use of one individual cable per electrode, hence, making the setup bulky and highly vulnerable to movement artefacts, particularly when the number of electrodes is increased. 

The use of screen-printed electrodes for high-density EMG recording has been recently presented in [19] as a case study for biopotential measurements during dynamic activities. The authors designed a matrix with 32 electrodes, using solid plastic pads placed below the conductive pads to ensure good contact with the skin and a sewed flexible PCB to interface the amplifier. In this work, we presented an extensive analysis of the applicability of screen-printed electrodes in a functional scenario for online myoelectric control. Moreover, as an alternative to the use of external elements to finalise the electrode design, our solution presented here does not require further steps after the screen-printing process is completed. The soft conductive polymer described in Section 2.1.1 provides stable skin-electrode contact during the execution of hand gestures. Additionally, the primer layer applied in the first stage of the printing process (Figure 2) provides the cotton fabric with enough strength and consistency to fit the electrode terminals securely into a ZIF connector, without the need for an additional interface between the electrode and the amplifier. The only external elements present in our setup were the extension flat cables (Figure 7c) that can be simply substituted by longer printed tracks. This will be implemented in future work. Moreover, the high fidelity of muscle activity signals recorded using TEX and the intrinsic adaptability of the interface to the stump after a traumatic amputation could potentially benefit lower-limb amputees using EMG-driven powered prostheses. Active prostheses are already commercially available, and EMG control of those systems is in the focus of recent research efforts [39]. The interface presented in this study could be an attractive choice for these applications as the soft materials are comfortable for long-term wear. This might be particularly important for users of lower limb prostheses, as their stump must support their body weight inside the socket, and solid components (i.e., rigid electrodes) are more likely to produce discomfort. 

Most of the designs proposed in the literature are not easily transferable for assembly-line production, which is important for the wider use of the interface in clinical and consumer applications. While the prototype presented in this study was produced mainly to benchmark the technology, the design and production processes are flexible and scalable. Screen printing is used throughout the textile manufacturing industry, and, thus, the inks and methodology used in this work can be easily transferred to the production line. In this case, the patterns were printed on a separate textile, but for the commercial solution, only a single fabric would be used and the electrodes printed as a set. This also highlights the benefit of a screen-printed electrode structure compared with weaving, knitting or embroidery because the pattern is not limited to orthogonal designs such as weaving and is only present on the side of the fabric it is required, unlike both knitting and embroidery. Similarly, the wiring pattern would be easily reconfigured to allow the printed tracks from each electrode set to be concentrated and allow a more robust and permanent interconnect. This process is the subject of future work. To validate the clinical potential of the technology, further studies will be conducted to test the feasibility of the dry electrode garment in amputees wearing advanced prosthetics as well as in patients suffering from sensory-motor disabilities for which clinical application of myocontrol could be beneficial (e.g., stroke).

## 5. Conclusions

We have presented a screen-printed textile electrode (TEX) for high-density EMG recording coated with a soft, dry, conductive polymer (IDUN DRYODE™) as electrode-skin interface. To benchmark this technology, we produced a similar electrode using a state-of-the-art approach (PET with hydrogel). The analysis of the recorded signals showed that PET and TEX ensure similar recording quality as well as the spatial distribution of muscle activity maps. The functional assessment demonstrated that our solution can be used for online myoelectric control in healthy participants and a person with a transradial amputation with the same performance as the benchmark technology. The proposed textile electrode is, therefore, an attractive solution for general purpose human-machine interfacing as well as clinical applications, where fast and simple setup is a critical requirement. 

## Data Availability

Data and materials can be made available upon reasonable request to the authors.
